# Sending a Message: Use of mRNA Vaccines to Target the Tumor Immune Microenvironment

**DOI:** 10.3390/vaccines11091465

**Published:** 2023-09-07

**Authors:** Fabiola Ramirez, Angelica Zambrano, Robert Hennis, Nathan Holland, Rajkumar Lakshmanaswamy, Jessica Chacon

**Affiliations:** 1 Paul L. Foster School of Medicine, Texas Tech University Health Sciences Center El Paso, El Paso, TX 79905, USA; fabiola.ramirez@ttuhsc.edu (F.R.); angelica.zambrano@ttuhsc.edu (A.Z.); rhennis@ttuhsc.edu (R.H.); nathan.holland@ttuhsc.edu (N.H.); rajkumar.lakshmanaswamy@ttuhsc.edu (R.L.); 2 L. Frederick Francis Graduate School of Biomedical Sciences, Texas Tech University Health Sciences Center El Paso, El Paso, TX 79905, USA

**Keywords:** cancer vaccines, mRNA, combination therapy, immunotherapy, dendritic cell, tumor immune microenvironment

## Abstract

While cancer immunotherapies have become central to treatment, challenges associated with the ability of tumors to evade the immune system remain significant obstacles. At the heart of this issue is the tumor immune microenvironment, the complex interplay of the tumor microenvironment and the immune response. Recent advances in mRNA cancer vaccines represent major progress towards overcoming some of the challenges posed by deleterious components of the tumor immune microenvironment. Indeed, major breakthroughs in mRNA vaccine technology, such as the use of replacement nucleotides and lipid nanoparticle delivery, led to the vital success of mRNA vaccine technology in fighting COVID-19. This has in turn generated massive additional interest and investment in the platform. In this review, we detail recent research in the nature of the tumor immune microenvironment and in mRNA cancer vaccines and discuss applications by which mRNA cancer vaccines, often in combination with various adjuvants, represent major areas of potential in overcoming tumor immune microenvironment-imposed obstacles. To this end, we also review current mRNA cancer vaccine clinical trials.

## 1. Introduction

Immunotherapy has revolutionized the world of cancer treatment. While chemotherapy, radiation therapy, and surgery are still the mainstays of cancer treatment, advances in immunotherapy consistently demonstrate superior outcomes [1,2] Nowadays, with proper immunotherapy treatment, patients with advanced-stage melanoma can achieve a five-year overall survival rate in approximately 50% of cases [3,4]. The considerable increase in the five-year overall survival rate in patients treated with ipilimumab has paved the way for the development of other immune checkpoint inhibitors (ICIs), such as programed cell death protein-1 (PD-1) and programmed cell death protein ligand-1 (PD-L1) inhibitors [3,4]. Despite the success of these immunotherapeutic agents, the development of resistance by way of tumor evasion of the immune system is a limitation of these novel treatments.

Tumor cells display antigens that can facilitate recognition by host CD8+ T cells. However, it is the avoidance of the host’s anti-tumor immune response that allows for the growth and dissemination of cancer cells. Much research describes the mechanisms by which tumors evade the immune system and the role of immune cells in creating an immunosuppressive niche for tumor cells to escape recognition. The importance of the immune components within the tumor microenvironment, also known as the tumor immune microenvironment (TIM), and antigen recognition within the tumor microenvironment (TME) has been highlighted by studying how cancer cells can avoid immune recognition. Similarly, evidence suggests that the presentation of cancer cell antigens to T cells differs from that of mature antigen-presenting cells (APCs). Costimulatory molecules must provide a second signal to activate the T cell response. Inadequate T cell activation or anergy can occur without a second signal, resulting in an inappropriate antigen-specific T cell response. Eliciting robust adaptive immune responses after successfully activating T and B cells is achieved by adequately delivering target antigens to the immune cells. Cancer vaccines offer an advantage in this scenario, as different vaccine formats can accomplish the targeted delivery of antigens. However, novel immunotherapy cancer vaccines must consider the complexity of the TIM. Understanding the interaction between immune cells and cancer cells is essential to the development of effective cancer vaccines.

The notion of cancer vaccines, which involves the activation of the innate immune response as a protective mechanism against cancer, is not new. In 1893, William Coley was the first to report on using mixed toxins to treat malignant tumors [5]. Similarly, in 1976, the intravesicular application of Bacillus Calmette–Guerin (BCG) proved superior to other standard chemotherapeutics in treating superficial bladder cancer [6]. Though the treatment of bladder cancer with BCG has been successful for over 30 years, the mechanism behind this successful immunotherapy is not well understood [7].

**Figure 1:**
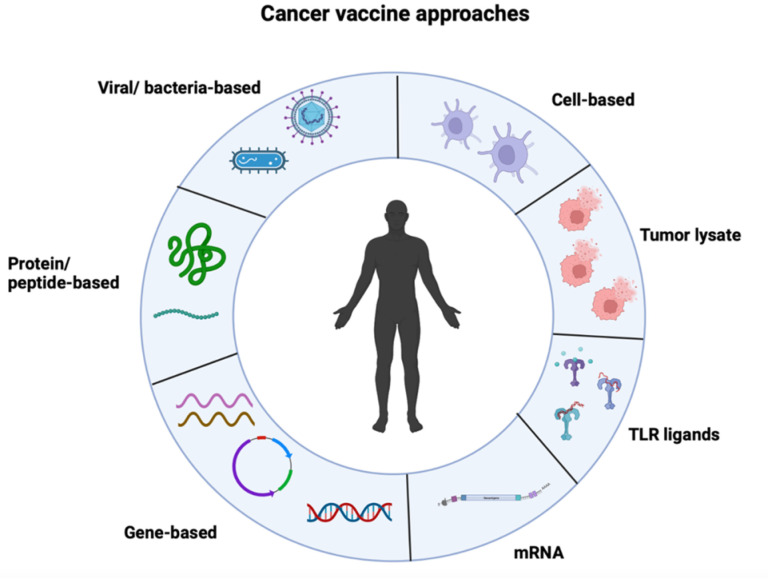
Several approaches may be used to administer cancer vaccines. These approaches can include mRNA, gene-based, peptide-based, viral/bacterial-based, cell-based, tumor lysate, or toll-like receptor (TLR) ligands. Each approach offers a unique set of benefits and limitations—including efficacy, side effects, safety, cost, and mode of delivery.

Cancer vaccines offer an alternative to the limitations posed by other forms of immunotherapy, but their limited success in clinical trials has shifted the focus towards other forms of immunotherapy. New advances in cancer vaccines have led to the development of targeted therapies against tumor antigens, peptides, and other tools, such as nucleic acids and viral vectors (Figure 1). As such, these tools fall within three major categories of cancer vaccines: nucleic acid vaccines, peptide vaccines, and cell vaccines. Our review article will primarily focus on mRNA cancer vaccines.

Nucleic acid vaccines, particularly mRNA cancer vaccines, have gained popularity due to the high efficacy of the COVID-19 mRNA vaccine [8]. Their potential to induce a strong humoral and cell-mediated immune response, as well as ease of production, make mRNA vaccines an attractive alternative to other forms of immunotherapy. After several years of research and clinical trials, the FDA has recently approved the first mRNA cancer vaccine as an adjuvant treatment for melanoma [9]. Herein, we highlight recent technological advances in mRNA vaccine technology, limitations of this form of immunotherapy, and opportunities for combination therapy. Furthermore, we present a detailed review of the TIM, a critical determinant of the success or failure of cancer immunotherapy.

## 2. mRNA Vaccines

Messenger RNA (mRNA) was discovered in 1961 by two groups including Sydney Brenner and James Watson [10,11]. In 1978, Gimitriadis showed that rabbit mRNA could be transported by liposomes into mouse cells and translate rabbit globin using the mouse cell’s ribosomes [12]. The liposomal delivery of exogenous mRNA was realized in human cells in vitro in 1987 and in vivo in 1990 [13]. By the 1990s, mRNA was widely recognized as a potential vaccine platform.

There are two general mRNA vaccine platforms, non-replicating and self-amplifying. While self-amplifying mRNA vaccines encode for the antigen of interest plus the RNA genome of a single-stranded RNA virus, allowing for RNA replication, non-replicating mRNA vaccines encode only the antigen of interest [14]. In the realm of cancer vaccines, only non-replicating mRNA vaccines are used [15].

The benefits of mRNA vaccines are numerous. Firstly, they exhibit a superior safety profile compared with other therapeutics, such as live attenuated and viral vector vaccines, both of which can confer potential pathogenicity [16]. mRNA alone does not contain the replicative machinery to multiply like a viral vector, rendering pathogenesis impossible. Secondly, while DNA-based vaccines can be toxic due to their stability or mutagenic due to their capacity to insert into host genomes, mRNA has fewer of these problems [17]. Although it is generally believed that the likelihood of reverse transcription is close to zero, it has been noted that some transpositional activity is seen throughout the genome due to endogenous elements or through reverse transcriptase shed by previous viral infections [18,19].

As cancer vaccines, mRNA vaccines are cost-effective and relatively easy to produce compared to other cancer therapeutics due to mRNA vaccine production’s modular nature [20]. Their rapid development can be advantageous when attempting to address specific mutations in cancer cells. Furthermore, as we make strides in the realm of personalized medicine, mRNA cancer vaccines provide flexibility and allow for customization based on the specific characteristics of each person’s cancer. Additional advantages of mRNA cancer vaccines reflect the relative cost and standardization profile that have emerged due to the massive investments brought to bear on mRNA production infrastructure during the SARS-CoV-2 pandemic. Most importantly, with optimal tumor antigens, mRNA cancer vaccines are likely to show superior efficacy [21]. mRNA vaccines can instruct cells to produce antigens with unparalleled precision [22]. Compared with whole-cell vaccines, which may take the immune system in any number of directions, mRNA vaccines enable researchers to target the exact antigen or antigens of interest. Furthermore, mRNA cancer vaccines can also provide a better immune response compared with short peptide vaccines, as peptides can bind in an unwanted fashion to human leukocyte antigen (HLA) class I molecules, disrupting effective molecular binding and blunting the desired immune response [23]. With the continuous development of other immunotherapeutic agents for the treatment of cancer, mRNA cancer vaccines can potentially be used as combination therapy, allowing other cancer treatments such as immune checkpoint inhibitors or targeted therapies to enhance their effectiveness.

As discussed in this review, addressing the multiple challenges that the TIM poses is essential to the fight against cancer and the successful application of any immunotherapeutic treatment. Unlike other forms of cancer therapy, mRNA vaccines can reshape the TIM in unique ways. For example, a study by Kreiter et al. demonstrated that neoantigen-based mRNA vaccines counteracted the immunosuppressive aspect of the TIM, ultimately resulting in efficient tumor control in vivo [24]. The success of the mRNA COVID-19 vaccines has in turn encouraged an explosion in further research on the mRNA delivery platform, notably in its potential use in cancer prophylactics and therapeutics. Challenges to implementing this technology have emerged primarily in undesired immunogenicity and adequate delivery. Despite the challenges that they face, mRNA vaccines were developed with exceptional speed and effectiveness during the COVID-19 pandemic [8].

### 2.1. Overcoming Unwanted Immunogenicity

Several obstacles exist in the development and clinical application of mRNA cancer vaccines. First, a significant challenge lies in overcoming immunogenicity. mRNA vaccines aim to spur the immune system to fight a specific pathogen or tumor, but inadequately prepared mRNAs animate innate immune cells, leading to systemic toxicity. Unaltered, in vitro transcribed (IVT) mRNA is recognized by pattern recognition receptors (PRRs) on APCs in the innate immune system [22], particularly toll-like receptor (TLR) -7 and -8. The activation of TLRs causes the release of pro-inflammatory cytokines such as type I interferons [25]. Other PRRs that recognize exogenous mRNA include retinoic acid-inducible gene-I-like and oligoadenylate synthetase receptors and RNA-dependent protein kinase, which can degrade mRNA before protein translation [26]. There may be some potential benefit of mild innate immune stimulation before intracellular antigen translation due to the early recruitment of CD8+ T cells to the area of interest. However, this still needs to be clarified and, even if true, must be heavily tuned to create the most efficacious balance.

There are multiple potentially undesirably immunogenic components of the mRNA structure. These include its cap, 5′ and 3′ untranslated regions (UTRs), poly(A) tail, and cytosine and guanine content. Perhaps most important is the actual chemical structure of mRNA nucleotides, particularly uridine [27]. Natural uridine acts as a ligand for TLR-7 and -8, promoting unwanted inflammation and decreasing mRNA translation [28]. In addition, natural cytosine is immunogenic. Landmark work from Karikó, Weissman, and others showed that chemically modifying uridine and cytosine decreases their undesirable immunogenicity while maintaining human ribosome translation [29]. Uridine nucleotides are replaced with methyluridine, 2-thiuridine, 5-methoxyuridine, or pseudouridine to combat immunogenicity. Cytosine is replaced with 5-methylcytidine. The replacement of adenosine with N1-methyladenosine or N6-methyladenosine has also been investigated as a potential improvement [30].

Recent studies have found that mRNA vaccines’ sequences can be integrated into human cells in vitro and in vivo [31,32]. While the likelihood of genome integration is believed to be low, the findings from these studies might account for the persistence of PCR-positive tests even after clinical recovery has occurred [32]. Although the exact mechanisms for the adverse events associated with the COVID-19 vaccine are not clear, genome integration offers another possible explanation. While the mRNA vaccines are overall considered to be safe, there have been severe adverse events reported following COVID-19 vaccination. These adverse events include myocarditis, pericarditis, thrombosis, and thrombocytopenia, among others [33]. More studies are needed to understand the mechanism behind genome integration and changes in gene expression, as well as close monitoring of vaccine-induced adverse events.

Another possible mechanism behind mRNA vaccine-induced adverse events is the unwanted immune system activation associated with unprocessed single-stranded mRNA (ssRNA) and its propensity to bind to other ssRNA molecules to form dsRNA. This form of dsRNA has been found to be highly immunogenic and structurally conserved by many viruses. In theory, vaccines using ssRNA have the potential to trigger excessive or uncontrolled immune responses, potentially leading to severe immune-related complications. The immunogenicity resulting from the spontaneous creation of dsRNA species during IVT is attenuated by decreasing the Mg^2+^ concentration, producing mRNA at elevated temperatures, or utilizing cellulose powder–dsRNA binding with fast protein liquid chromatography [26,34,35]. However, the perhaps most promising approach to inhibiting the formation of dsRNA is solid-state techniques, currently being developed, that can completely eliminate dsRNA contamination and provide complete sequence specificity.

### 2.2. Targeting and Bioavailability

For effective use, mRNA vaccines must target cells, tissues, and organs with sufficient bioavailability for effective translation. Additionally, mRNA vaccines must undergo endocytosis by appropriate cells for cellular entry. Even if mRNA were able to avoid toxigenic PRR interaction, naked mRNA degrades too rapidly for adequate bioavailability, resulting from its susceptibility to nucleophilic attack and hydrolysis through the presence of a 2′ hydroxyl group, contributing to hydrogen bonding instability. Moreover, naked mRNA is attacked by the immune system and destroyed. To overcome these limitations, researchers have developed various delivery devices to protect mRNA during transit to the target cell cytosol. Among these are lipid nanoparticles (LNP); besides protecting mRNA directly from degradation and aiding in endosomal escape, they are scalable and quickly produced [36]. LNPs were developed initially to transport small interfering ribonucleic acids. Four components comprise an LNP: a phospholipid, an ionizable cationic lipid, a lipid-linked polyethylene glycol (PEG), and cholesterol [22]. The phospholipid maintains the bilayer structure of the nanoparticle, ionizable cationic lipids (ICL) allow the release of mRNA molecules from endosomes into the cytosol, and PEG and cholesterol are used as stabilizers. Each of these components can be fine-tuned for maximal antigen delivery and expression. The proper charge profile and shape of ICLs are paramount. For example, appropriate ICLs generally have a pKa between 6 and 7. LNPs are assembled at low pH, which protonates the ICL. This allows its inclusion in the LNP, where they bind to mRNA and helper phospholipids [37]. At physiologic pH, ICLs are deprotonated and thus contribute to LNP neutrality, which is needed to prevent toxicity. Once imported into acidic cellular endosomes, ICLs protonate, which enables them to bind with negative phosphate groups on endosomal membranes to disrupt the endosome and release the mRNA into the cytosol [38]. Recently published work by Suzuki et al. reports the development of novel ionizable cationic lipids that increase immunogenicity and decrease the need for cold storage [39]. The ICL shape is an essential factor for the endosomal release of mRNA vaccines. The canonical or cone-shaped form is generally achieved by desaturating ICL lipid tails or, in more recent years, using branched ICL lipid tails. The ICL charge profile is also essential because it can determine the organ localization of the LNP. Kranz et al. [25] demonstrated that highly cationic charge distribution results in the lung accumulation of LNPs, whereas a negative charge leads to distribution in the spleen or liver [40]. The development of LPN structures appropriate for different applications and target organs is an area of ongoing research.

### 2.3. Delivery

The injection method and site are major considerations in effective mRNA vaccine delivery. Several delivery methods currently exist, including intramuscular, intradermal, subcutaneous, intravenous, and intratumor or intranodal [41]. Intramuscular injections, the current standard for mRNA SARS-CoV-2 delivery, offer an optimal balance of large-quantity vaccine delivery, diminished injection site reactions, and the recruitment of diverse APCs [42]. Intradermal vaccines also provide an effective delivery route due to the robust presence of Langerhans cells and macrophages within the dermis [41]. Pardi et al. demonstrated that the intramuscular and intradermal delivery of mRNA LNPs resulted in the most prolonged translation duration [43]. The recruitment of diverse subsets of APCs within the muscle may be attributed to the highly vascular network within muscle tissue.

As another form of delivery, intravenous injection allows for the substantial delivery of the mRNA load directly to the peripheral lymph nodes and lymphatic organs but carries an increased risk of toxicity [36]. Despite this risk of peripheral toxicity, the intravenous delivery of mRNA is superior for the induction of a robust cytotoxic CD8+ T cell response [25]. Subcutaneous injection into the subcutaneous fat allows for large quantities of mRNA delivery. Additionally, subcutaneous delivery methods are associated with few adverse side effects and injection site reactions [44]. Intratumoral and intranodal injections permit significantly smaller doses of vaccine but offer substantial delivery efficacy. Important work by Thielemans et al. demonstrated the promising use of intranodal delivery, which has the potential to activate CD8a and dendritic cells near the tumor site and in the surrounding lymph [45]. They also demonstrated significant antigen translation at the injection site and, crucially, within CD11c+ cells in draining lymph nodes [45]. While intratumoral and intranodal delivery methods are more direct, they pose significant challenges due to percutaneous lymph node or tumor access.

### 2.4. mRNA Vaccine Clinical Development

As previously noted, the research and development of mRNA vaccines have exploded over the past several years. While there are different formulations of mRNA vaccines, some of the most common include nanocarrier systems containing lipids or peptides. The development of such formulations prevents the degradation of the mRNA by extracellular ribonucleases (Rnases) and allows for the facilitated uptake of the mRNA by APCs. In protamine-formulated mRNA vaccines, the mRNA is packaged with protamine to reduce degradation [46]. The mRNA with protamine combination results in enhanced protein expression and immunogenicity. This combination, known as the RNActive vaccine, has been evaluated in several clinical trials. According to ClinicalTrials.gov, NCT03164772 and NCT00923312 are the most recently completed clinical trials examining the effectiveness of RNActive against non-small-cell lung cancer (Table 1). NCT03164772, which examined the efficacy of RNActive plus combination immunotherapy, reported a median progression-free survival of 2 months with the mRNA vaccine + durvalumab alone and 1.8 months with vaccine + durvalumab + tremelimumab. Results have not been reported for trial NCT00923312.

Dendritic cells are powerful APCs capable of mounting an effective anti-tumor response. Their role in stimulating cancer-specific T cell responses has been well studied [47]. Within the context of cancer vaccines, DCs are often used to prime a patient’s immune system against cancer cells. While this is typically done by loading DCs with tumor-specific peptides, a newer mRNA transfection strategy is quickly gaining enthusiasm. This strategy consists of the ex vivo manipulation of DCs and loading them with mRNA encoding a desired tumor antigen. As of 22 February 2023, a total of 22 clinical trials examining the effectiveness of mRNA-loaded DC vaccines have been completed (Table 1). Out of the 22 completed clinical trials, only two have reported results. The effect of nivolumab alone or in combination with mRNA pulsed DCs was evaluated in a phase 1 trial for the treatment of glioblastoma (NCT02529072). The DCs were pulsed with human cytomegalovirus pp65-lysosomal-associated membrane protein (pp65-LAMP) mRNA for this study. The results of this study, which enrolled a total of six subjects, documented similar grade 1–2 adverse effects, such as fatigue and thrombocytopenia, for both arms of the study. Grade 4 adverse effects such as wound infection and meningitis were recorded for three subjects in the combination therapy arm of the study. According to the authors, this study was terminated early due to results from the CheckMate 142 phase 3 trial that did not demonstrate improved survival with nivolumab alone for recurrent glioblastoma [48].

In study NCT02366728, the impact of pre-conditioning (unpulsed vs. human CMV pp65-LAMP mRNA pulsed) on the migration of DCs was evaluated in patients with a diagnosis of glioblastoma that had undergone resection and had completed standard temozolomide and radiation treatment. The study also examined the impact of pre-conditioning with tetanus toxoid and basiliximab on survival. According to the authors, this confirmatory study’s results corroborated the effect of pre-conditioning with tetanus toxoid on the enhanced migration of DC vaccines to the draining lymph nodes [49]. More studies evaluating the route of delivery as well as the level of migration of DCs are of critical importance, as data suggest that DC vaccines delivered intradermally demonstrated limited migration to lymph nodes [50].

The success of mRNA vaccines depends heavily on a number of factors, including the ability to induce a robust anti-tumor immune response. The treatment of cancer, including the development and use of mRNA cancer vaccines, has been challenging for a myriad of reasons. The ability of tumor cells to evolve and evade the host’s immune system, known as “cancer immunoediting”, creates unique challenges in the war against cancer [51]. Cancer cells thrive within a complex network of immune cells and stromal components [52]. This stromal and cancer cell network varies in composition within the tumor and between patients with the same tumor histology. In addition, the TIM, consisting of immune cells, cytokines, and tumor cells, plays a key role in cancer. The interplay between the immune cells and cancer cells within the TIM determines the effect of anti-tumor immunity. We will discuss the role of the TIM in more detail, specifically the components that factor into pro-tumor and anti-tumor immunity.

### 2.5. The Role of the Tumor Immune Microenvironment in Cancer Progression and Regression

**Figure 2:**
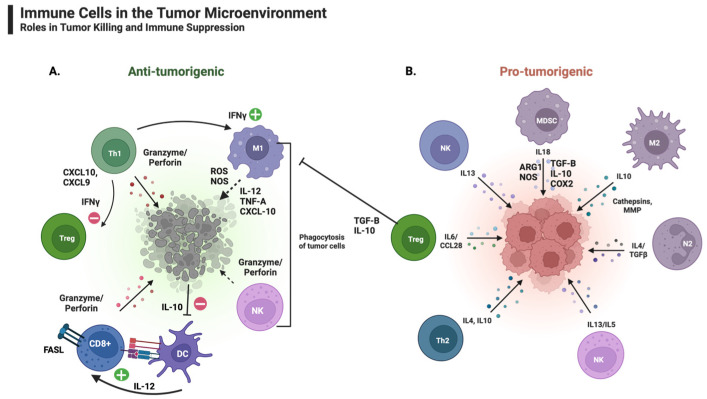
The TIM consists of tumor cells, immune cells, and cytokines. The interplay between the pro- and anti-tumorigenic properties within the TIM determines the tumor progression or regression. (**A**) M1 macrophages demonstrate anti-tumor effects via direct cytotoxicity and antibody-dependent cell-mediated cytotoxicity. Furthermore, TH1 T cells can downregulate Treg differentiation via the secretion of IFN-γ. (**B**) In a pro-tumorigenic environment, adaptive immune responses support the activation of several pathways. The activation of DCs is inhibited by cytokines such as IL-10, hindering the initiation of the adaptive immune system. Similarly, the infiltration of tumors by Tregs leads to the suppression of both adaptive and innate immune responses. The release of cytokines such as IL-10 and TGF-β supports the propagation of Tregs within the TIM. Similarly, M2 macrophages contribute to tumorigenesis by promoting tumor invasion, dissemination, and angiogenesis by generating various cytokines, including IL-1, IL-8, TNF-α, MMP-9, MMP-2, and VEGF.

The anti-tumorigenic immune components of the TIM (Figure 2A) include DCs, lymphocytes, natural killer cells (NKs), and M1 macrophages [53,54]. These cells are crucial to arrest tumor development, particularly in the early stages of tumor growth [54]. In contrast to these cells, immunosuppressive or pro-tumorigenic cells (Figure 2B) within the TIM include myeloid-derived suppressor cells (MDSCs), regulatory T cells (Tregs), tumor-associated neutrophils (TANs), and type 2 polarized tumor-associated macrophages (M2) [53]. The immune components of the TIM vary in composition and number based on the stage of tumor development [54].

The stromal component of the tumor consists of macrophage lineage cells, fibroblasts, vascular endothelial cells, and the extracellular matrix. Besides providing structural support for tumor growth, tumor stromal cells have been found to affect the infiltration of immune cells [53]. The inactivation of fibroblast-activating protein, a marker of tumor-associated fibroblasts, has been explored as a possible approach to anti-cancer therapy.

Triggered by tumor antigens, the innate and adaptive immune systems respond by releasing cytokines and chemokines, leading to chronic inflammation within the TIM. Chronic inflammation due to infection, autoimmune diseases, and obesity contributes to the induction of oncogenic mutations and local immunosuppression [55,56]. Once genetic alterations lead to oncogenesis, transcription factors such as nuclear factor kappa B (NF-κB) and STAT3 activate the expression of genes encoding inflammatory cytokines, inducible nitric oxidase synthase (iNOS), angiogenic factors, adhesion molecules, and enzymes in the prostaglandin synthesis pathway [56].

Several mechanisms link these inflammatory cytokines with the role of tumor initiation, promotion, malignant conversion, invasion, and metastasis [55,56,57,58]. For example, it has been proposed that instead of exerting a normal immunoprotective mechanism, tumor-infiltrating lymphocytes (TIL) within the pro-inflammatory TIM may induce genomic instability by enhancing the rate of molecular mutations via the generation of reactive oxygen species (ROS) and reactive nitrogen species (RNS) and may weaken anti-tumor immunity [58,59]. Within the TIM, the types of immune cells and their precise location, function, and quantities can all influence a tumor’s response to treatment. Furthermore, the crosstalk between tumor immune cells can lead to an immunosuppressive and pro-angiogenic tumor environment [54,58]. A delicate balance must exist between the anti-tumorigenic and pro-tumorigenic components within the TIM, which can determine the outcome of the anti-tumor immune response.

With regard to the development of successful mRNA cancer vaccines, it is critical to understand how the TIM influences various aspects of the vaccine’s response and efficacy. For example, in a general overview, mRNA cancer vaccines work by delivering tumor-specific antigens to APCs, which in turn present these antigens to T cells. The role of each cell within the TIM, including each cell’s activation status, influences how well the antigen presentation process occurs. Understanding which antigens are more optimal for an effective immune response within the TIM, as well as targeting the right APCs within the TIM, is necessary for the successful activation of T cells.

Additionally, as we make progress in the realm of personalized medicine, understanding and targeting the unique characteristics of each individual’s TIM is crucial in tailoring mRNA cancer vaccines. Having a clear understanding of the TIM can ultimately lead to the identification of tumor-specific antigens that are most likely to provoke and effective immune response in everyone. Furthermore, mRNA cancer vaccines are a new avenue for the introduction of ICIs, and it is essential to understand the TIM’s immunosuppressive microenvironment for the successful development of such sequences. The TIM is intricately connected to the effective development of mRNA cancer vaccines. A TIM’s composition, including immune cell interactions and the level of immunosuppression, can influence an mRNA cancer vaccine’s ability to stimulate a proper immune response. Understanding the role of immune cells and their interactions within the TIM is critical to developing successful immunotherapeutic agents.

## 3. Pro-Tumorigenic Immune Cells and Factors within the TIM

### 3.1. Neutrophils

Within the TIM, tumor-associated neutrophils (TANs) play a significant role in tumor biology and cancer progression and represent an important negative prognostic marker for various cancers. Unlike naïve neutrophils, TANs can be classified as anti-tumorigenic (N1) or pro-tumorigenic (N2) [60]. Pro-tumorigenic neutrophils can lead to the establishment of a premetastatic niche, ultimately contributing to tumor angiogenesis, growth, and metastatic dissemination [60]. Whether an N1 or N2 phenotype develops within the tumor cells depends on regulatory cytokines, such as transforming growth factor-beta (TGF-β), which drive differentiation of the TANs towards the N2 phenotype [61]. Relatedly, blocking TGF-β favors the differentiation of N1 TANs [61]. The critical role of TGF-β in the regulation of TAN differentiation has led to the development of TGF-β blockers and TGF-β receptor inhibitors to prevent tumor spread [61,62].

### 3.2. Macrophages

Similar to TANs, tumor-derived chemokines are vital in recruiting monocytes to the TIM. Once inside the TIM, monocytes differentiate into tissue-resident macrophages. Tumor-associated macrophages (TAMs) are an integral part of the TIM. Functions of TAMs within the TIM include tumor growth, invasion, metastasis, and drug resistance [58,63]. TAMs are of two functionally different types, M1 and M2 macrophages, posing pro-inflammatory or immune-suppressive effects, respectively. Both types have a great degree of plasticity, and changes within the TIM influence a state of constant differentiation [63]. M1-type macrophages have anti-tumor effects. They directly mediate cytotoxicity as well as antibody-dependent cell-mediated cytotoxicity [63]. Furthermore, M1 macrophages are activated by naïve FN-γ and characterized by an elevated ability to secrete cytokines such as IL-1β, TNF-α, IL-12, and MHC class II molecules [64]. As demonstrated by various studies, pro-inflammatory cytokines can paradoxically have both pro-tumorigenic and anti-tumorigenic roles [65]. Within the TIM, the anti-tumorigenic effect occurs in part due to the pro-inflammatory cytokines’ ability to increase tumor cell apoptosis and suppress various inflammatory elements within the TIM, such as ROS, iNOS, and MMPs [65].

M2-type macrophages, in contrast, are considered pro-tumorigenic. Their role in tumor metastasis is involved in the production of soluble factors such as matrix metalloproteinases (MMPs), serine proteases, and cathepsins, which ultimately lead to the degradation of the tumor’s matrix membrane and tumor cell invasion and dissemination [63]. Additionally, M2 macrophages are involved in the promotion of angiogenesis by way of tissue remodeling and vascularization. Releasing several cytokines, including IL-1, IL-8, TNF-α, MMP-9, MMP-2, and VEGF, achieves tissue remodeling and vascularization [56,63,64].

The effect of TAMs on immune regulation, including leukocyte recruitment and survival, shows that TAMs can directly inhibit CD8+ T cell proliferation and recruit Tregs via CC-chemokine ligand 22 (CCL22) [64,66]. Chemokines (CCL2 and CCL5) and cytokines, such as colony-stimulating factor-1 (CSF-1), can lead to the recruitment of inflammatory monocytes to the TIM. Of particular importance is CSF-1; elevated levels of CSF positively correlate with poor cancer prognosis [56,63,67]. Similarly, NF-κB has been found to polarize the balance between M1- and M2-type macrophages towards the M2 phenotype [56]. Gordon et al. demonstrated that PD-1, an immune checkpoint receptor that is upregulated in activated T cells, is also expressed by TAMs in the TIM [68]. The success of PD-1 and PD-L1 blockade in cancer therapy rests on the fact that tumor cells and TAMs tend to overexpress PD-L1, resulting in immune system inhibition. Although the mechanism by which PD-1/PD-L1 blockade leads to T cell activation is well known, further studies are needed to understand the direct role of anti-PD-1/PD-L1-blocking antibodies on TAMs and other immune cells within the TIM.

Tumors exist as “hot” or “cold” based on their immune cell landscape in the TIM. Generally, cold tumors, also known as immune deserts, do not respond to immunotherapy, whereas hot tumors do, due to their infiltration of immune cells. In theory, TAMs, specifically M2 phenotypes, residing along tumor margins prevent cytotoxic lymphocyte infiltration into the tumor core, resulting in a poorly immunogenic or cold tumor [69]. Targeting TAMs as a cancer treatment strategy remains an active area of investigation.

### 3.3. Myeloid-Derived Suppressor Cells

Myeloid-derived suppressor cells (MDSCs) are bone marrow-derived cells that share a common progenitor with TAMs. The production of chemokines, such as CXCL5 and CXCL12, leads to the attraction of MDSCs to the TIM [56]. MDSCs suppress the body’s adaptive immune response to tumor cells, inhibiting T cells and NKCs within the TIM by expressing arginase, inducible NOS, TGF-β, IL-10, and COX2, as well as by increasing the local Treg population, among others [70]. Furthermore, MDSCs can lead to metastasis and tumor proliferation via the downregulation of STAT3 and the production of VEGF and other essential mediators of tumor angiogenesis [56,63,70].

### 3.4. Regulatory T Cells

Tregs suppress adaptive and innate immune responses and play a central role in maintaining immunologic tolerance. By inhibiting IFN-γ secreted by CD8+ T cells, Tregs aid the proliferation and maintenance of an M2-type macrophage-dominant TIM and a direct immunosuppressive effect results from the expression of surface molecules such as CTLA-4 and the secretion of cytokines (IL-10, TGF-β). Given the potent suppression of the immune-mediated tumor response by Tregs, targeting this cell population presents another approach to cancer therapy. For example, tumor-specific Tregs exhibit many cell surface markers, such as CTLA-4 and OX40 [54]. Targeting tumor-specific Treg cell surface markers with antibodies effectively established a systemic anti-tumor immune response capable of eradicating metastasis in mice [71]. Similarly, targeting CTLA-4 with monoclonal antibodies improved overall survival in patients with metastatic melanoma [1,2]. Recently, combination therapy targeting the action of both CTLA-4 and PD-1 has been suggested as a more effective approach than monotherapy for various solid tumors.

### 3.5. mregDCs

DCs play a complex role in the TIM. DC1s are potent antigen presenters and are critical in priming the responses of CD4+ and CD8+ T cells. Unlike B cells and macrophages, DCs activate naïve T cells and induce isotype switching in B cells without T cell help [72]. Although they have the potential to mount an effective immune response against cancer cells, the TIM can lead to impaired DC function, resulting in the suppression of immune responses, which further facilitates tumor growth and progression. For example, tumor cell-secreting cytokines such as TGF-β and IL-10 have been shown to suppress DC function. Furthermore, tumors can upregulate immune checkpoint molecules, such as PD-L1, inhibiting DC function and limiting T cell activation.

While DCs are essential to a successful immune response, a subset of DCs within the TIM influences the T cell response. Initially identified by Maier et al., mregDCs regulate T cell responses, both negatively and positively [73]. These DCs express immunoregulatory and maturation genes and arise from DC1s and DC2s [73]. The capacity of mregDCs to negatively regulate the T cell response arises from the initiation of receptor tyrosine kinase AXL-dependent PD-L1 upregulation. In contrast, the blocking of IL-4 signaling is thought to increase the immunogenicity of mregDCs, resulting in positive T cell effector function [73,74]. The treatment of mregDCs with an IL-4 blockade might become another avenue for cancer treatment. Regarding cancer vaccines, determining whether a DC is tolerogenic vs. immunogenic is essential to avoid mregDCs’ activation upon vaccination.

## 4. Anti-Tumorigenic Immune Cells and Factors within the TIM

### 4.1. T Cells: Th1

T cell interactions within the TIM greatly influence tumor survival and cancer progression. The release of cytokines plays an integral role in the activation and differentiation of pro-inflammatory Th1 T cells or anti-inflammatory Th2 T cells [75]. Within the TIM, naïve T cells can be polarized to differentiate into Th1 T cells via the release of IL-12 [76]. TH1 T cells mediate efficient tumor cell lysis via the potent recruitment and activation of CD8+ cytotoxic T cells through IFN-γ. Furthermore, Th1 T cells also promote NK cytotoxicity via IL-2 secretion [77]. NKs and CD8+ T cells serve as potent tumor immune defenses within the TIM via enhanced tumor cell lysis. Th1 T cells may also amplify tumor immune defenses via the recruitment of cytotoxic leukocytes and the secretion of chemokines, including CXCL10 and CXCL9 [78]. The potent induction, recruitment, and amplification of cytotoxic leukocytes by Th1 T cells, therefore, aids in sustaining high levels of anti-tumor activity within the highly immune-suppressive milieu of the TIM. Furthermore, Th1 T cells also promote anti-tumor immunity by activating APCs via co-stimulatory molecules. The activation of APCs allows the further enhancement of immune-mediated tumor cell identification and subsequent neoplastic cell lysis. While Th1 T cells have demonstrated a strong affinity to promote antigen presentation and the enhanced induction of cytotoxicity within the TIM, speculation exists that Th1 T cells downregulate immune-suppressive Tregs through IFN-γ secretion [79]. Furthermore, CD4+ Th1 T cells potently inhibit the accumulation of MDSCs through the TNF-related apoptosis-inducing ligand (TRAIL) pathway [80].

### 4.2. T Cells: Th17

Th17 T cells protect against extracellular microbes such as bacteria and fungi and drive the pathogenesis of several autoimmune diseases. Within the TIM, the role of Th17 T cells remains less clear due to the ability of Th17 to demonstrate either pro-tumor or anti-tumor responses depending on the unique tumor environment [81]. Th17 cell induction occurs within the TIM via the production of cytokines IL-6, TGF-β, and IL-1β locally [82]. Th17 may function to promote tumorigenesis through the secretion of its hallmark cytokine IL-17. Within the TIM, IL-17 promotes angiogenesis through the potent induction of VEGF and PGE2 [83]. Angiogenesis allows for the efficient delivery of oxygen and nutrients to the rapidly dividing tumor cells. Furthermore, IL-17 enhances tumorigenesis via the secretion of IL-6. IL-6 enhances STAT3 pro-tumor signaling [84]. Conversely, Th17 cells can also act as potent inhibitors of tumorigenesis. Th17 cells promote tumor immunity via the induction of CXCL9 and CXCL10, key chemokines that promote the infiltration and activation of cytotoxic NK cells and CD8+ T cells within the TIM [85]. The conflicting pro-tumor and anti-tumor functions of Th17 T cells highlight the need for more robust research surrounding the polarization and function of Th17 T cells within the TIM.

### 4.3. T Cells: TH2

While Th2 T cells are recognized for their integral role in mediating allergic reactions and parasitic infections [86], Th2 T cells also play a dual role within the TIM, serving both pro- and anti-tumor functions. Th2 T cells are potent secretors of the cytokines IL-4, IL-5, 1L-10, and IL-13. While cytokines like IL-4 and IL-5 are most well recognized for their role in mediating type I immediate hypersensitivity reactions, these cytokines may also influence tumor development. The role of Th2 T cells in the promotion of tumorigenesis is currently attributed to a variety of immune-suppressive mechanisms. Among these mechanisms are the potent secretion of IL-10, a cytokine that is well recognized for its prominent role in immune suppression through the inactivation of Th1 T cells and mitigation of Th1 polarization. Furthermore, IL-10 has also been demonstrated to impair neoplastic antigen processing and presentation on MHC I to CD8+ T cells [87]. Conversely, Th2 secretion of IL-5, an integral mediator of eosinophil activation, has been demonstrated to promote eosinophil and macrophage tumor infiltration and destruction. Within the TIM, eosinophils are speculated to function in concert with other tumoricidal myeloid cells to inhibit tumor cell proliferation [88]. This has been demonstrated within mice models, as IL-5-deficient mice display decreased eosinophil concentrations within the TIM and the subsequent loss of anti-tumor immune function [88]. Additionally, IL-4 may also play a prominent role in inhibiting tumorigenesis via the promotion of the polarization of TAMs into cytolytic M1 macrophages within the TIM [89].

### 4.4. CD8+ T Cells

CD8+ T cells are well recognized to be a cornerstone of the immune-mediated killing of virally infected and neoplastic cells. Induced through the secretion of IL-12 from APCs, CD8+ T cells mediate effective cellular killing via the secretion of perforin, granzyme, IFN-γ, cathepsin, and TNF-α [90]. CD8+ T cells mediate effective cytotoxicity via binding to MHC I on the surfaces of infected cells. Subsequently, CD8+ T cells may secrete perforin and granzyme, which create holes within the cellular membrane and activate intracellular caspases, respectively. Opposingly, CD8+ T cells may also utilize the FAS ligand (FAS-L) to bind to FAS receptors on target cells to induce the activation of caspases and subsequent apoptosis. While these components promote tumor cytotoxicity, CD8+ T cells’ secretion of IFN-γ plays a multifocal role in mitigating tumorigenesis. For example, IFN-γ upregulates the tumor cell expression of MHC I, directly promoting CD8+ T cell recognition and cytotoxicity [91]. Furthermore, IFN-γ has also been demonstrated to “reprogram” and inactivate Tregs in order to mitigate their immune-suppressive effects [92]. For these reasons, elevated IFN-γ secreting CD8+ T cells within the TIM has been associated with a more favorable prognosis within several cancer types [93,94,95,96].

### 4.5. Natural Killer Cells

NK cells represent a central component of the TIM. NK cells are characterized by a CD3-CD56+/CD16+ phenotype. NK cells are regulated by the expression and subsequent binding of activating and inhibitory receptors to their respective ligands. Upon the binding of activating receptors, such as NKG2D, NKp30, NKp44, and NKp46, to their ligands, NK cells can exhibit cytotoxic abilities by secreting granzymes and perforin [97,98]. Unlike CD8+ cells, which kill their targets in an MHC-dependent manner, NK cells are able to lyse their targets independently of MHC I expression [97,98]. Tumor cells try to evade NK cell lysis by expressing ligands that bind to inhibitory receptors, such as killer Ig-like receptor (KIR) or CD94/NKG2A on the NK cells [97,98]. Various approaches to harnessing NK cell activation within the TIM have been explored. For example, the monoclonal antibody Monalizumab (an NKG2A-blocking antibody) has been demonstrated to enhance NK cell activity and subsequent anti-tumor activity in some clinical trials [98].

### 4.6. NKT Cells

Natural killer T cells (NKT) are a subset of CD1-d-restricted T cells that possess both characteristics of NK cells and T cells depending on the unique TIM. NKT cells may participate in tumorigenesis and anti-tumor activity by influencing leukocyte polarization. For example, Th1 subtype NKT cells are potent inhibitors of tumor cell growth and differentiation by producing Th1 cytokines IFN-γ and TNF-α [99]. The secretion of these cytokines by Th1-like NKT cells triggers the induction and activation of nearby CTLs and NK cells for cytotoxic-mediated tumor killing. Furthermore, Th1-like NKT cells also can induce the apoptosis of M2 macrophages within the TIM and promote the polarization of the M1 phenotype via GM-CSF, further inhibiting tumorigenesis [100]. Opposingly, NKT cells can polarize into immune-suppressive Treg NKT or Th2 NKT subtypes. Shifts towards this unfavorable phenotype may result from the overstimulation of NKT cells during tumor development, resulting in anergy. Treg NKT cells predispose patients to tumor growth and progression via the inhibition of T cell function through the secretion of IL-10 and TGF-β [101]. Furthermore, Treg-like NKT cells may enhance the polarization and induction of M2 TAMs via the potent production of IL-10 [102].

### 4.7. Dendritic Cells

DCs are vital components in both innate and adaptive immunity within the TIM. DCs participate in tumor antigen presentation and recognition and have a prominent role in the secretion of co-stimulatory factors and polarizing cytokines [103]. Generally, the TIM favors the production of tolerogenic DCs via the secretion of the cytokines TGF-β, IL-10, and indoleamine-pyrrole 2,3-dioxygenase (IDO) [103]. Tolerogenic DCs are specialized to induce immune suppression by differentiating naïve CD4+ T cells into regulatory T cells. Tregs are a particular class of T cells that promote anergy and peripheral tolerance. While most Tregs are generated within the thymus during negative selection, DCs within the peripheral tissue play an integral role in the induction of Tregs within the TIM.

Whereas tolerogenic DCs suppress immune responses within the TIM, select immunogenic DCs mitigate tumor growth and metastasis. Conventional dendritic cells 1 (cDC1), a sub-type of conventional DCs, demonstrate efficacy in tumor antigen presentation and activating CD8+ T cell responses within the lymph nodes draining the tumor [104]. cDC1 secrete large amounts of IL-12, which promotes the polarization and activation of CD8+ cytotoxic lymphocytes for the perforin- and granzyme-mediated killing of tumor antigens. Furthermore, cDC1 promotes tumor destruction via potent interplay with local NK cell populations. cDC1 potentiates NK cell function via the secretion of IL-12 and IFN-γ, which support the recruitment and activation of NK cells within the TIM [105]. In opposition to cDC1, cDC2 can present antigens on MHC II and activate CD4+ T cells [106]. Another special subclass of DCs, the plasmacytoid DC (pDC), is capable of orchestrating anti-tumor activity through the secretion of type 1 interferon (IFN-1) in response to neoplastic cells [107]. Furthermore, these pDCs may also direct anti-tumor immunity via antigen presentation via MHC II, although their efficacy as APCs is debatable [108]. Interestingly, several recent studies have added to the body of evidence that pDCs within the TIM are poorly immunogenic—as pDCs offer limited efficacy in carrying out IFN-1-mediated tumor killing [109]. pDC expression of tolerogenic factors such as IL-10, TGF-β, and IDO supports pDC pro-tumor immunity [103]. For this reason, high pDC tumor infiltration may indicate a worse prognosis in several cancer types [110,111].

## 5. Combination Approaches That Target the TIM and Utilize mRNA Vaccines for Cancer Therapy

While traditional cancer treatment modalities are still necessary, immunotherapy provides an important armamentarium for the treatment of advanced or metastatic cancers. Cancer immunotherapy aims to eliminate cancer cells by activating the host anti-tumor immunity. There have been various approaches developed over the years to accomplish this, with this review article primarily focusing on the efforts of mRNA vaccines. The COVID-19 pandemic and the subsequent COVID-19 mRNA vaccine development helped to redirect the focus of mRNA vaccines not only towards disease treatment and prevention but cancer treatment. The efficacy of mRNA vaccines for cancer therapy can largely rely on the TIM. As outlined in the previous section, the TIM contains both pro- and anti-tumor properties. While the TIM presents a significant challenge to effective monotherapy, combination therapy may lead to successful manipulation of the TIM. Targeting the TIM’s immune-suppressive components in combination with mRNA vaccines can be a promising therapeutic avenue to combat cancer [112]. Current research efforts focus on immunotherapy combination approaches; by exploiting the benefits of multiple immunotherapeutic agents, numerous combination therapy clinical trials have demonstrated significant improvements in overall responses [113,114,115,116].

As scientific advances in the stabilization and optimization of mRNA delivery progress, more mRNA vaccines are being utilized as a part of combined immunotherapy treatments. Several clinical trials utilizing mRNA vaccines in combination with different checkpoint inhibitors have demonstrated potent anti-tumor effects. In contrast, while these results are promising, the use of mRNA vaccines as monotherapy does not appear to confer similar efficacy. In trials assessing the use of mRNA vaccine monotherapy for the treatment of non-small-cell lung cancer, mRNA vaccine monotherapy did not appear to confer a significant difference in overall median survival compared to maintenance chemotherapy [117]. The limited efficacy of mRNA vaccine monotherapy may be attributed to the highly immunosuppressive milieu of the TIM or the requirements for the frequent administration of a vaccine with extremely high potency [118]. As discussed previously, some of these immunosuppressive factors within the TIM include inhibitory cytokines like IL-10 and TGF-β, which favor the differentiation of tolerogenic DCs. Additional immunosuppressive factors within the TIM include strongly angiogenic cytokines like IL-17, which promote local nutrient and oxygen delivery through VEGF.

Although mRNA cancer vaccines may have limitations as standalone immunotherapeutic agents, when combined with other agents, they have the potential to become crucial to effective cancer treatment. mRNA cancer vaccines in combination with other immunotherapeutic agents, particularly ICIs, can lead to an enhanced immune response against cancer cells. Vaccines prime the immune system, while the ICIs unleash and amplify the immune response. Although numerous clinical trials are still in their early phases, encouraging results have led to the groundbreaking FDA Therapy Designation of a personalized mRNA cancer vaccine, mRNA-4157/V940, in combination with pembrolizumab for the treatment of high-risk melanoma following complete resection. This approval, granted on 22 February 2023, was based on positive data from a phase 2b trial (NCT03897881) and will allow investigators to move forward with a phase 3 trial. This momentous milestone demonstrates the tremendous therapeutic potential of cancer vaccines, particularly mRNA cancer vaccines. As shown in Table 1 and Table 2, nine completed clinical trials and three currently recruiting trials have examined the effect of combination therapy with mRNA-based cancer vaccines.

## 6. Conclusions

The tumor microenvironment plays an integral role in the influence and polarization of both pro-tumor and anti-tumor immune defenses. Therefore, the immune cells within the TIM can be utilized to develop highly potent and specific cancer immunotherapies. Of these emerging immunotherapies, mRNA vaccines have demonstrated promise to promote efficacious anti-tumorigenic immune responses within the TIM.

The benefits of mRNA vaccines may be attributed to the unique ability of mRNA to promote tumor antigenicity via the induction of both cell-mediated and humoral immune responses. These mRNA immunotherapies have demonstrated significant promise due to their scalability, low cost, and high specificity. The primary challenges associated with mRNA vaccines include minimizing the inherent immunogenicity of mRNA while also optimizing the adequate delivery of mRNA to the TIM. While these challenges have limited the use of these cancer vaccines as standalone treatments, numerous ongoing clinical trials have demonstrated substantial efficacy in minimizing the tumor burden. Therefore, continued efforts to investigate mRNA vaccines and their therapeutic benefits, as well as the TIM, are imperative to the development of successful immunotherapeutic agents against cancer.

## Figures and Tables

**Table 1:** mRNA cancer vaccines with a completed status.

Trial Status	Type of Cancer	mRNA Vaccine Type	Vaccine Route	Combination Therapy	NCT Number
Completed	Metastatic Non-Small-Cell Lung Cancer	RNActive	intradermal	in combination with either durvalumab or with durvalumab + tremelimumab	NCT03164772
Completed	Non-Small-Cell Lung Cancer	RNActive	not recorded	none	NCT00923312
Completed	Glioblastoma	mRNA-based DCs	not recorded	none	NCT02808364
Completed	Glioblastoma	mRNA-based DCs	intradermal	in combination with chemo/radiotherapy + temozolomide	NCT02709616
Completed	Malignant Glioma/Astrocytoma/Glioblastoma	mRNA-based DCs	intradermal	in combination with nivolumab	NCT02529072
Completed	Hematological Malignancies	mRNA-based DCs	intravenous	none	NCT02528682
Completed	Glioblastoma	mRNA-based DCs	intradermal	in combination with temozolomide +/− basiliximab	NCT02366728
Completed	Melanoma	mRNA-based DCs	intradermal	alone or in combination with cisplatinum	NCT02285413
Completed	Multiple Myeloma	mRNA-based DCs (LCs)	subcutaneous	none	NCT01995708
Completed	Melanoma	mRNA-based DCs	intranodal	none	NCT01530698
Completed	Melanoma	mRNA-based DCs (LCs)	subcutaneous	none	NCT01456104
Completed	Prostate Cancer	mRNA-based DCs	intradermal	in combination with docetaxel	NCT01446731
Completed	Malignant Melanoma	mRNA-based DCs	intradermal or intranodal	in combination with IL-2	NCT01278940
Completed	Prostate Cancer	mRNA-based DCs	not recorded	none	NCT01278914
Completed	Melanoma	mRNA-based DCs	intradermal, intravenous	none	NCT01066390
Completed	Breast Cancer, Malignant Melanoma	mRNA-based DCs	intradermal	in combination with cyclophosphamide	NCT00978913
Completed	Glioblastoma Multiforme	mRNA-based DCs	intradermal	none	NCT00890032
Completed	Glioblastoma	mRNA-based DCs	intradermal	none	NCT00846456
Completed	Acute Myeloid Leukemia	mRNA-based DCs	intradermal	none	NCT00834002
Completed	Glioblastoma Multiforme	mRNA-based DCs	not recorded	in combination with radiotherapy, temozolomide, basiliximab	NCT00626483
Completed	Acute Myelogenous Leukemia	mRNA-based DCs	not recorded	none	NCT00510133
Completed	Melanoma Stage III or IV	mRNA-based DCs	not recorded	none	NCT00243529
Completed	Liver Metastases from Colorectal Cancer	mRNA-based DCs	intradermal, intravenous	alone	NCT00228189
Completed	Malignant Melanoma	mRNA-based DCs	intradermal	in combination with GM-CSF	NCT00204516

m-RNA cancer vaccine clinical trials with a “completed” status were found on ClinicalTrials.gov using the search terms “cancer”, “mRNA”, and “vaccine” on 22 February 2023. DCs: dendritic cells; LCs: Langerhans cells.

**Table 2:** mRNA cancer vaccines currently recruiting.

Trial Status	Type of Cancer	mRNA Vaccine Type	Vaccine Route	Combination Therapy	NCT Number
Recruiting	EBV+ Malignant Tumors	not reported	intramuscular	none	NCT05714748
Recruiting	Adult Glioblastoma	mRNA lipid nanoparticle	intravenous	after standard radiation treatment	NCT04573140
Recruiting	Advanced Solid Tumors	mRNA neoantigen	subcutaneous	none	NCT05198752
Recruiting	Prostate Cancer	mRNA lipoplex	intravenous	alone or in combination with cemiplimab	NCT04382898
Recruiting	Solid Tumors	mRNA lipid nanoparticle	intramuscular	alone or in combination with pembrolizumab	NCT03313778

mRNA cancer vaccine clinical trials with a “recruiting” status were found on ClinicalTrials.gov using the search terms “cancer”, “mRNA”, and “vaccine” on 22 February 2023.

## Data Availability

Not applicable.

## Abbreviations

APC	Antigen-Presenting Cell
BCG	Bacillus Calmette–Guerin
cDC	Conventional Dendritic Cell
CCL22	CC-Chemokine Ligand 22
CD	Cluster of Differentiation
COX	Cyclooxygenase
CSF-1	Colony Stimulating Factor-1
CTLA	Cytotoxic T-Lymphocyte-Associated protein
CXCL5	C-X-C Motif Chemokine 5
DC	Dendritic Cell
DNA	Deoxyribonucleic Acid
dsRNA	Double-Stranded Ribonucleic Acid
ECM	Extracellular Matrix
FAS-L	FAS Ligand
FDA	Food and Drug Administration
HLA	Human Leukocyte Antigen
ICI	Immune Checkpoint Inhibitors
ICL	Ionizable Cationic Lipids
IDO	Indoleamine-Pyrrole 2,3-Dioxygenase
IFN-I	Type I Interferon
IL	Interleukin
inOS	Inducible Nitric Oxidase Synthase
irAEs	Immune-Related Adverse Events
IVT	In Vitro Transcribed
KIR	Killer Ig-Like Receptor
LC	Langerhans Cells
LN	Lymph Node
LNP	Lipid Nanoparticle
M1	Type 1-Polarized Macrophages
M2	Type 2-Polarized Macrophages
MDSC	Myeloid-Derived Suppressor Cell
MHC	Major Histocompatibility Complex
MMP	Matrix Metalloproteinases
mregDCs	Mature DCs Enriched in Immunoregulatory Molecules
mRNA	Messenger RNA
NK	Natural Killer Cells
NKT	Natural Killer T Cells
NO	Nitric Oxide
NF-b	Nuclear Factor kappa B
OS	Overall Survival
PEG	Polyethylene Glycol
PD-1	Programed Cell Death Protein-1
PD-L1	Programmed Cell Death Protein Ligand-1
pDC	Plasmacytoid Dendritic Cell
pp65-LAMP	pp65-Lysosomal-Associated Membrane Protein
PRR	Pattern Recognition Receptor
ROS	Reactive Oxygen Species
RNase	Ribonuclease
RNS	Reactive Nitrogen Species
siRNA	Small Interfering Ribonucleic Acid
ssRNA	Single-Stranded Ribonucleic Acid
STAT3	Signal Transducer and Activator of Transcription 3
TAM	Tumor-Associated Macrophages
TAN	Tumor-Associated Neutrophil
TCR	T Cell Receptor
TGF-β	Transforming Growth Factor-Beta
Th1	T Helper 1 Cells
Th2	T Helper 2 Cells
TIM	Tumor Immune Microenvironment
TLR	Toll-Like Receptor
TME	Tumor Microenvironment
TRAIL	TNF-Related Apoptosis-Inducing Ligand
Tregs	Regulatory T Cells
TIL	Tumor-Infiltrating Leukocytes
UTR	Untranslated Region
VEGF	Vascular Endothelial Growth Factor
